# Successful bridge to recovery using two-stage HeartWare LVAD explantation approach after embolic stroke

**DOI:** 10.1186/1749-8090-8-233

**Published:** 2013-12-30

**Authors:** Anton Sabashnikov, Benjamin A Högerle, Prashant N Mohite, Aron-Frederik Popov, Diana García Sáez, Javid Fatullayev, Mohamed Amrani, Thorsten Wahlers, André R Simon, Toufan Bahrami

**Affiliations:** 1 Department of Cardiothoracic Transplantation & Mechanical Support, Royal Brompton and Harefield NHS Trust, Harefield Hospital, Hill End Road, Harefield, Middlesex, United Kingdom UB9 6JH; 2 Department for Cardiothoracic and Vascular Surgery, University of Göttingen, Robert-Koch-Strasse 40, 37075, Göttingen, Germany; 3 Department of Cardiothoracic Surgery, Heart Center, Univesity Hospital of Cologne, Kerpener Strasse 62, 50937, Cologne, Germany

**Keywords:** Left ventricular assist device (LVAD), LVAD thrombosis, Stroke, Bridge to recovery

## Abstract

A two-stage explantation of a left ventricular assist device (LVAD) was performed on 47 year old afro-american gentlemen with non-ischemic dilated cardiomyopathy (DCM) who was successfully bridged to recovery. After he suffered a stroke caused by a VAD thrombosis with embolisation, the VAD outflow graft was first ligated using minimally-invasive approach. Two months later, the device was explanted and a manufactured titanium plug was placed into the sewing ring. This stepwise procedure might be beneficial in cases of high thromboembolic risk and in patients who suffered a thromboembolic event previously.

## Background

Ventricular assist devices (VADs) provide an established mechanical support of patients with end-stage heart failure as bridge to transplantation, bridge to recovery and chronic support [1].

However, successful LVAD explantation can only be performed in a restricted number of patients who meet the criteria of myocardial recovery [2,3]. In this respect, our institution has reported encouraging results with pharmacological therapy combined with continuous-flow or pulsatile-flow LVADs as bridge to recovery [2,3].

The new miniaturized centrifugal HVAD® Pump (HeartWare Inc, Framingham, MA) is a novel third generation VAD. One of the most significant design advantages of the HeartWare HVAD Pump is its size, intrapericardial placement and versatility in terms of implant options [4]. Due to the miniaturized dimensions it can be implanted over a minimal invasive approach without sternotomy [5].

Full sternotomy LVAD explantation is shown to be feasible; however it might be associated with increased risk of intraoperative bleeding, need for blood transfusions and postoperative ventricular dysfunction. This problem might be partially resolved by LVAD explantation via minimally invasive lateral thoracotomy [6]. However, in cases of high thromboembolic risk, both approaches may lead to embolic stroke due to manipulation and handling of LVAD during surgery. In this report, we present our experience with staged LVAD explantation in a patient with myocardial recovery who suffered an embolic stroke following LVAD thrombosis. This approach was used with the view to preventing potential flush of clots from the thrombosed LVAD reducing the risk of further potential stroke.

## Case presentation

A 47-years-old afro-american gentleman diagnosed with non-ischemic dilated cardiomyopathy (DCM) underwent a continuous-flow LVAD (HeartWare) implantation as a bridge to transplantation. Postoperatively, the device flow was 6.6 L/min, left ventricular ejection fraction (EF) was 16%, and the left ventricular systolic (LVESD) and diastolic (LVEDD) diameters were 63 and 71 mm, respectively. After an uncomplicated postoperative course, the patient was discharged home. Six month later, his cardiac parameters improved significantly (LVESD: 26 mm, LVEDD: 46 mm, EF: 63%). The exercise test according to Modified Bruce Protocol [3] revealed that the maximum volume of oxygen (VO_2_ max) on support was almost constant at 2,700 rpm and 1,800 rpm. A transthoracic echocardiogram (TTE) confirmed no serious heart dilatation after 15 min of exercise. Due to these evident signs of myocardial recovery the patient was admitted for an elective LVAD removal.

**Figure 1:**
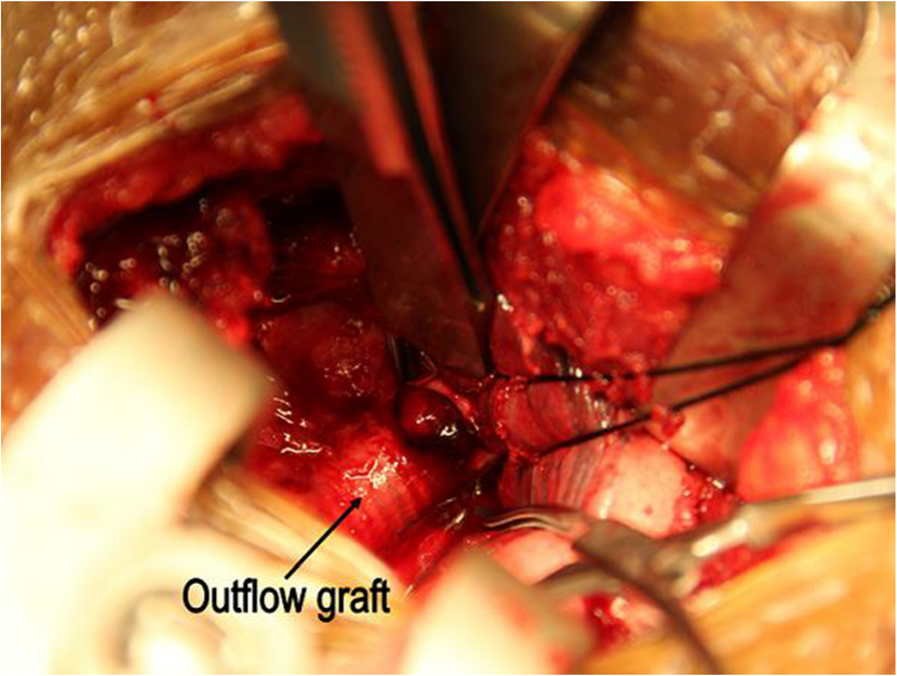
**Access to the outflow graft through a miniaturized incision right parasternally at the level of the third intercostal space**.

**Figure 2:**
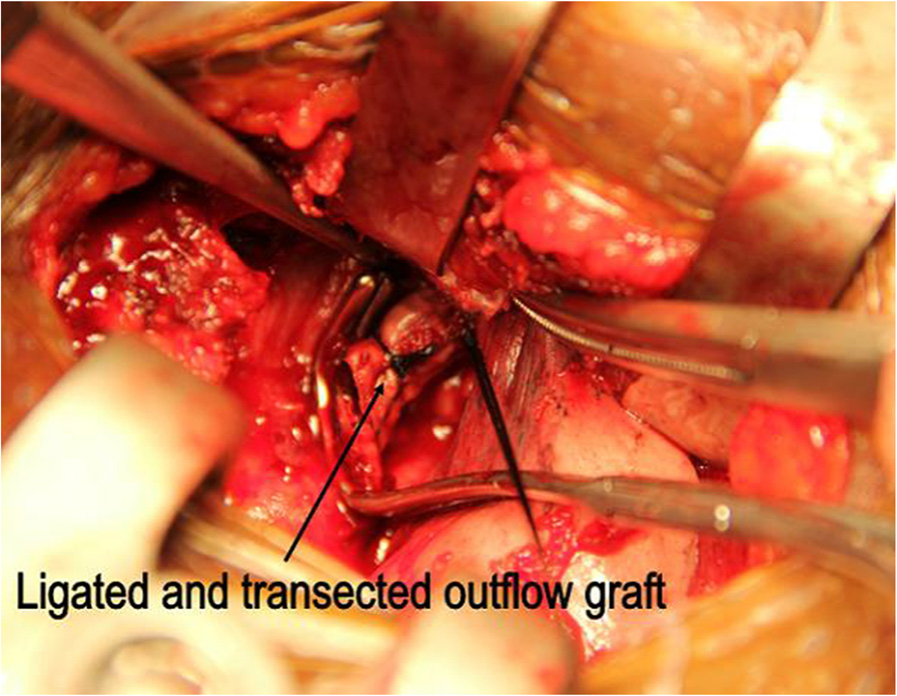
**The outflow graft was ligated and transected**.

Unfortunately, he suffered a right fronto-parietal lobe infarct a night before the elective operation. The main areas of neurological impairment were left arm weakness, language and attention. Suggesting a thrombotic formation in the device and a displaced thrombus causing the stroke, three days after this event the LVAD was stopped; the outflow graft (Figure 1) was ligated and cut in between (Figure 2). This was done through a miniaturized incision right parasternally at the level of the third intercostal space. As clots occluding the outflow graft were confirmed intraoperatively, the distal part of the outflow graft was inspected and remaining clots were removed. Subsequently, the patient participated in a post-stroke rehabilitation program with progressive recovery.

After two months, the LVAD pump was electively explanted through median sternotomy in off-pump technique. A manufactured titanium plug was placed into the inflow sewing ring which was left in place. Postoperative examination of the pump confirmed a dense thrombus formation with a soft red thrombus cast around the impeller of the device. With a quick postoperative recovery, the patient was discharged home with stable condition, satisfactory cardiac parameters and improving neurological status. From the myocardial recovery point of view, there have been no acute heart failure symptoms, no orthopnea and no peripheral oedema over 6 months follow-up.

## Discussion

The two-stage LVAD explantation in patients with myocardial recovery and high risk of thromboembolic events offers several advantages compared to the conventional approach. The ligature of the VAD outflow graft through a small intercostal incision might prevent thrombotic flush from the VAD which can occur during extensive dissections during conventional LVAD explantation. Also, patients who already suffered an embolic stroke might benefit from additional time for recovery from previous stroke before LVAD is explanted. Furthermore, such a stepwise approach might reduce intra- and postoperative complications, such as right ventricular dysfunction and bleeding with the need for massive transfusions.

In our case, the sewing ring of the LVAD was not removed but was closed using a manufactured titanium plug. As previously described, this approach may reduce the risk of perioperative bleeding and infections [7]. Also, the operating time is shortened due to no need for explantation of the ring and closing the left ventricle. It also preserves the geometry of the left ventricle.

Previous research describes thrombolysis also as a suitable treatment of VAD thrombosis. However, performed in emergency and in the setting of thrombolysis and anticoagulation introduced for VAD thrombosis, it may lead to severe bleeding complications. Furthermore, in our particular case, the VAD explantation was the best option due to recovery of myocardial function.

The risk of perioperative complications might be minimized even more if the VAD explantation is also performed without cardiopulmonary bypass [6]. This technique might reduce the risk of infection, hemodynamic instability, and can be done without complete mobilization of the heart. However, the feasibility of this approach should be considered in individual cases dependent on topographical location and accessibility of the LVAD.

## Conclusion

The two-stage VAD explantation is a well reasonable option in cases of high thromboembolic risk and in patients who suffered a thromboembolic event previously.

## Consent

A written informed consent was obtained from the patient’s next of kin for publication of this case report and accompanying images. A copy of the written consent is available for review by the Editor-in-Chief of this journal.

## Competing interests

The authors declare that they have no competing interests.

## Authors’ contribution

AS wrote the manuscript; BH, PNM and AFP co-wrote the manuscript and managed figures; DG and JF were involved in the concept, TW, MA, TW, and ARS made critical revision of the manuscript, TB approved the manuscript. All authors read and approved the final manuscript.
